# Antimicrobial Resistance and Use on Chinese Dairy Farms: Awareness and Opinions Regarding Selective Treatments of Farm Managers

**DOI:** 10.3390/antibiotics13090854

**Published:** 2024-09-06

**Authors:** Yindi Xiong, Herman W. Barkema, Jingyue Yang, John P. Kastelic, Diego B. Nobrega, Xiaoping Li, Xiaofang Tong, Zhenying Fan, Jian Gao

**Affiliations:** 1 College of Veterinary Medicine, China Agricultural University, Beijing 100193, China; yindi.xiong@ucalgary.ca (Y.X.); bs20213050473@cau.edu.cn (J.Y.); bs20243050615@cau.edu.cn (X.L.); s20223050981@cau.edu.cn (X.T.); 2 Faculty of Veterinary Medicine, University of Calgary, Calgary, AB T2N 4N1, Canada; barkema@ucalgary.ca (H.W.B.); jpkastel@ucalgary.ca (J.P.K.); diego.nobrega@ucalgary.ca (D.B.N.); 3 Xianwei CARE Technology Platform, Beijing 100191, China; xianwei202208@163.com

**Keywords:** antimicrobial stewardship, clinical mastitis, dry cow therapy, dairy farms, antimicrobial resistance

## Abstract

Background: In China’s expanding dairy industry, a lack of oversight regarding antimicrobial use and increasing antimicrobial resistance are evident. Selective treatments of dairy cows for clinical mastitis or dry cow therapy are proposed to promote judicious antimicrobial use without adversely impacting cattle health. These approaches have been successfully implemented on farms in other countries. Methods: On 28 October 2023, a 2-day in-person seminar was held in Beijing, China, on selective antimicrobial treatments of dairy cows for clinical mastitis or dry cow therapy on large Chinese dairy farms. Concurrently, a qualitative study involving 15 technical managers from the 13 largest Chinese dairy groups used focus group discussions and questionnaires to explore perspectives on selective treatments of dairy cows for clinical mastitis or dry cow therapy. The main outcomes assessed were opinions and concerns regarding implementing selective antimicrobial treatments. Results: Although there was diversity of cognition on AMR and selective treatments, the technical managers were generally positive regarding adoption of selective treatments. However, they expressed a need for more evidence and tools, including anticipated economic impacts, effects of delaying treatment until diagnosis, accurate interpretation of milk recording data, safe use of internal teat sealants, and spread of pathogens. Participants stressed the need for awareness, staff training, farm management, and China-specific standards, suggesting large-scale trials to assess efficacy of selective treatments. Conclusion: The findings revealed key challenges and barriers currently impeding selective AMU practices. These insights could inform efforts to promote judicious AMU on farms through targeted treatment regimens, reducing mounting selective pressure driving resistance.

## 1. Introduction

Antimicrobial resistance (AMR) is a major global public health threat in the 21st century, largely due to the overuse and misuse of antimicrobials in humans and food animals [1]. China has been the world’s second-largest consumer of antibiotics since 2010. The burden in human medicine of multidrug-resistant (MDR) infections in China is substantial, with an estimated 7.0 million inpatient human cases annually, substantially higher than the 2.8 million cases in the United States and representing a $77 billion economic burden [2].

Although AMR has been extensively studied in human medicine, substantial AMR and run-off of antimicrobials from animal husbandry into the environment represent an under-examined driver of AMR in much of the world. In 2013, China’s human population consumed 48% of the country’s antimicrobials, whereas the rest were used in animals [3]. This is concerning, as use and overuse of antimicrobials in animals can promote the emergence of drug-resistant bacteria [4]. Despite regulations intended to reduce inappropriate AMU (antimicrobial use) in human medicine [5], AMU in food animals is largely unsupervised in China.

China’s dairy industry is proliferating, with total raw milk production reaching 42.0 million tonnes in 2023—the sixth consecutive year of growth, according to the National Bureau of Statistics [6]—making China the world’s fourth largest dairy producer. Although dairy farming contributes less to the abundance of AMR genes than other livestock sectors, with the absolute abundance of AMR genes in cow manure being around three orders of magnitude lower than in swine and chicken [7], AMR still remains a concern in dairy settings in China. The prevalence of bacteria resistant to several antibiotics is much higher in China than in Western Europe, North America, Australia, and New Zealand, particularly among those that cause mastitis [8,9,10,11,12,13].

Most AMU on dairy farms occurs intramammarily for treating clinical mastitis (CM) or dry cow therapy (DCT) [14,15]. While administering antibiotics to all clinical cases and following dry-off procedures, as recommended since late 1960 by the National Institute for Research in Dairying, are effective in controlling contagious mastitis pathogens, environment pathogens like *E.coli* and *Klebsiella* spp. have become increasingly predominant [16]. Given the changing circumstances and the emerging threat of AMR, selective treatment protocols for non-severe clinical mastitis (STCM) and selective dry cow therapy (SDCT) have been proposed. The principle of selective treatment is to use antimicrobials only on cases that will benefit from the drug. For STCM, cows with mild/moderate clinical mastitis are sampled and cultured to determine the pathogen, with antimicrobials administered only to cases showing good sensitivity. In SDCT, antibiotics are withheld from cows that are healthy and less likely to have intramammary infections. Both selective treatment approaches have been tested on farms to reduce AMU without adversely impacting animal health [17,18]. However, there are apparently no reports of studies examining selective treatment protocols in China.

In October 2023, China Agriculture University, with support from the Xianwei CARE technology platform, a consultancy company for dairy farms, hosted an in-person, 2-day seminar in Beijing, China, on selective antimicrobial treatments for large Chinese dairy farms. Importantly, the seminar was attended by representatives from the largest dairy farms in China, which together produce a fifth of the country’s total milk output. This qualitative study had the following aims: (1) understand Chinese dairy farmers’ and veterinarians’ perception of mastitis-related AMU; (2) identify motivators and barriers to adoption of STCM and SDCT; and (3) provide suggestions for potential adoption of STCM and SDCT on large Chinese dairy farms. It is expected that insights gained will inform efforts to promote judicious AMU through evidence-based treatment strategies tailored to the Chinese dairy industry.

## 2. Results

All participants were responsible for veterinary management of multiple farms. The affiliated groups owned a total of 361 farms with ~1,562,600 cattle, comprising 26.4% of the total number of dairy cattle in China in 2021 (Table 1). Six of the thirteen companies each contributed >500,000 tons of raw milk and 12 had >10,000 kg average annual milk yield per cow in 2021.

### 2.1. Awareness of AMR

All participants were cognizant of the existence of AMR and its consequences in both human medicine and veterinary settings (Table 2). However, their opinions on AMR in animals and its relevance for humans varied. Although 62% of participants cared about AMR and its negative impacts on human health, they were not concerned about the impacts of farm AMU on the health of humans. As one participant mentioned, “I know we use a lot of antibiotics for farm animals, but with the mandatory withdrawal periods, I don’t think that we harm human health (Q1E2)”. Although not fully informed on the exact mechanism, some participants expressed openness to accepting current scientific evidence, with one stating “I have never heard before about how antibiotics in the environment can make bacteria resistant, but I believe research supports this (Q1D2)”. Therefore, we inferred that although awareness of AMR was high among the participants, perspectives on food-animal contributions to AMR transmission pathways remained mixed, with 62% of participants mentioning AMR genes and residuals (Table 2).

What was most concerning to participants is that AMR is present on dairy farms; 92% of participants thought the spread of AMR would damage product quality, and 85% agreed it would have an adverse impact on consumer acceptance (Table 2). Worsening the AMR situation would lead to a negative perception of the dairy industry. Other concerns included increased production costs, especially regarding bovine mastitis and including extra labor, cost of drugs, more milk discarded, etc. One participant shared his experience of resistant mastitis pathogens on farms: “One of our farms reported that the mastitis situation was getting worse, and they had to prolong antimicrobial treatments, but things were not getting better. There was resistance to the antibiotics they were using. However, after they switched from cefquinome to cefalexin, the situation improved (Q1B3)”. Direct experience with AMR challenges appeared to increase apprehension among stakeholders.

The companies of some participants performed antimicrobial susceptibility testing on mastitis pathogens in their central laboratory and they shared interesting findings regarding mastitis pathogen antimicrobial sensitivity profiles: “When we perform AMR testing on mastitis pathogens, some strains are resistant to third-generation cephalosporins but sensitive to first- or second-generation products (Q2C1)”. However, none of the companies represented routinely conducted AMR testing on mastitis pathogens for their farms. Another participant highlighted, “There are not many products to choose from if resistance emerges. Some farms will likely continue to use current products, even if decreased efficacy is suspected (Q1D1)”.

Despite mixed perspectives on potential public health impacts, all participants agreed that curtailing agricultural AMU is an emerging priority, and one stated, “Restricting antibiotic use is the main trend that the nation is expecting (Q1D3)”. Some also cited consumer perceptions and market influences driving reduced AMU, with one noting, “Our group has several organic farms and consumers of those products want us to minimize antibiotic use (Q2C1)”.

The lack of comprehensive AMR surveillance is a major obstacle, with one participant stating, “One of the biggest problems is that there is no national system for monitoring AMR profiles of agriculture-related bacteria (Q1A1)”. Robust AMR data was deemed essential for informing and developing responsible AMU policies.

### 2.2. Awareness of SDCT

All participants had some knowledge of SDCT. Regarding the source of information, one said, “The company that sells teat sealants told us all about that years ago, but it seems like they were just trying to sell more products (Q12A1)”. Introducing teat sealants also brought new knowledge on dry-period management to the Chinese dairy industry.

Experience with SDCT implementation varied, with 31% of participants reporting having tried SDCT on farms. However, poor results diminished enthusiasm in some, as one shared: “It is a lot of trouble to perform SDCT and the [clinical] mastitis rate increased during the next lactation. Therefore, we stuck to using blanket dry treatment of cows after that (Q13E3)”. Another participant stated “We tried SDCT before, but economically speaking, it did not save much money. It is just not worth doing that (Q14C3)”. Although one participant’s operation currently has an ongoing SDCT trial slated for a follow-up in April, none of the participating dairy companies routinely used SDCT.

### 2.3. Awareness of STCM

No farm of the participating companies currently used STCM, giving antibiotics to every CM case (Table 2). Awareness of STCM lagged that of SDCT, with only 38% of participants familiar with the concept of STCM (Table 2). For participants who never heard of STCM, entrenched views that withholding antibiotics is wrong challenged acceptance of STCM: “It would be hard to convince farm veterinarians that you do not have to use antibiotics to treat some cows [with CM] (Q3D1)”. Routine treatment, regardless of cause, is another hurdle, as another noted: “Most farms give antibiotics without considering culture results. When [clinical] mastitis occurs, they treat them promptly (Q3A3)”. Thus, beliefs that CM always warrants the use of antibiotics by default encouraged overuse without targeting likely pathogens.

### 2.4. Major Concerns for SDCT

Since SDCT experience varied, a peer-reviewed manuscript on SDCT [19], translated into Mandarin, was presented to the participants. Responses to this manuscript were analyzed to identify key themes related to challenging SDCT adoption, outlined in the subsections below.

#### 2.4.1. Milk Recording (DHI)

Most Chinese dairy companies participate in monthly DHI either internally or via contracted third-party evaluators. Standard Chinese DHI testing includes monthly tests, including estimating milk production and SCC for every cow, with 61% of participants reporting having DHI performed on all farms whereas others only performed it on some of their farms (Table 3).

Currently, only 31% of participants indicated that they use DHI data to adjust management. However, an important reason for not valuing DHI data was the lack of systematic analytical tools to interpret a large dataset; 69% of participants suggested the need for a standard protocol to analyze DHI data to harness the information. Furthermore, another consideration is timing. Most participants agreed, with one stating: “It is necessary to see the average of the latest 3 DHI test results to see the SCCs to understand overall udder health (Q18E1)”. While affirming the value in assessing lactation-wide SCC averages from multiple DHI tests, 31% of participants did not agree that the latest DHI should be used for selecting cows to perform SDCT. They stressed that “The process of sampling, sending samples to the lab and getting results, lasts forever. By the time we get results, dry cow therapy has already been done (Q19D2)”. They suggested, “It is better to use previous SCCs or a cow-side test to check udder health (Q19C1)”. Therefore, a timely response may suit the need for assessing IMI immediately before dry-off.

#### 2.4.2. Teat Sealants

In China, blanket DCT, including long-acting antibiotics and internal teat sealants, is standard procedure. Every farm group reported using teat sealants in dry cow management, although the exact number of farms was not reported (Table 3). The consensus among most participants was that teat sealants are an essential component of SDCT. One individual emphasized the importance of internal teat sealants by stating, “It must be used in SDCT. If you don’t use antibiotics and teat sealants in dry cows, you leave the teat canal open (Q23E3)”. However, 31% of individuals insisted that antibiotics are an essential component of drying-off (Table 3). One participant explained, “We are using teat sealants, but we believe it is the antibiotics that protect dry cows from new infections (Q22A3)”.

One of the main concerns regarding the use of teat sealants was residual sealants in the milk during the week after calving, which can be problematic (Table 3). As one participant suggested, “Sometimes we still find residuals of teat sealants in milk after removal (Q23D1)”. Inexperienced workers may mistakenly interpret the presence of residual sealant in milk as CM. Also, one stated, “Some farms reported cows occasionally dying rapidly soon after drying off; we did a necropsy and determined they had severe mastitis (Q23D2)”. They suspected that poor hygiene during administration of teat sealant introduced bacteria into the udder.

#### 2.4.3. Economics

In discussing economic benefits of selective treatments, one shared his experience, “We have tried SDCT, but it only saved 400,000 CNY (~US$57,000) in treatment costs, which is nothing for a farm with more than 10,000 cows (Q13C3)”. The perceived benefit did not align with the associated risks.

Moreover, the cost of teat sealants is another issue. Despite widespread use of teat sealants at drying-off on many farms, 92% of participants think current teat sealants are overpriced. One suggested, “We would gladly switch to more affordable products that deliver similar results if they were available (Q23A1)”.

### 2.5. Major Concerns Regarding STCM

To clearly outline the scientific background and detailed decision-making processes under an STCM approach, we presented a recent review [20], translated into Mandarin. Based on the workflow provided in the review, participants raised their concerns, which were summarized into topics below.

#### 2.5.1. Delaying Treatment 1 Day

Implementing STCM protocols requires basing AMU on bacteriological culture results for non-severe CM cases [20]. Therefore, cows cannot be treated immediately at first signs of CM, but laboratory culture results must be available. On most Chinese dairy farms, milkers make the initial diagnosis of CM based on clinical signs, often segregating suspect cases into another barn for veterinary evaluation and initiation of treatment within 12 h. However, to enable selective approaches, this diagnosis-to-treatment timeframe would need to be extended to accommodate culture-guided treatment decisions. A major apprehension raised by the participants was that delaying treatment could worsen mastitis outcomes, as one stated, “We have always been taught to detect and treat cases as early as possible. Intentionally leaving cows with mastitis untreated seems to contradict this (Q3D1)”. In addition, overcoming the instinct to immediately administer antimicrobials poses a challenge. Beyond individual-cow outcomes, participants also worried about the spread of pathogens within the herd, posing questions like “What if cultures come back as contagious pathogens like *Staph[ylococcus] aureus* or *Strep[tococcus] agalactiae*? Transmission risks seem high without prompt treatment (Q6C2)”. A participant suggested mitigating this concern by “using distinct leg bands on suspect CM cases or moving them to a separate barn so that veterinarians know to prioritize monitoring while awaiting culture results (Q6C3)”. This would, however, add to the workload.

Although they believed that the fastest possible intervention following diagnosis leads to better treatment outcomes, 92% of participants deemed it acceptable to keep CM cows untreated if valid data from trials on Chinese farms demonstrated it was not harmful (Table 4).

#### 2.5.2. Diagnostics

Current diagnostic methods used for udder health were reported by participants; 54% of companies used culture-based methods to diagnose mastitis pathogens, whereas 46% sent milk samples to third-party laboratories (Table 4). Furthermore, 31% of participants reported that they were using rapid diagnostic test tools, whereas 23% used only traditional culture methods (Table 4). As STCM requires rapid pathogen identification, rapid diagnostic tests would be useful.

Aseptic sampling is imperative for diagnostic accuracy. Contamination of samples was reported to affect >5% of samples for 61% of participants, whereas 23% stated that their farms did not regard contamination as a possibility of bacterial culture, which could lead to misinterpretations of pathogens (Table 4). A participant mentioned, “Contamination is also a problem. Sometimes, there are various pathogens on an agar plate (Q8E1)”. In addition, some farms did not list contamination as a diagnostic result, causing unreliability in reporting culture results (Table 4).

The interval between the first diagnosis and identifying the pathogen was another challenging concern for most participants. A participant suggested, “If we want to test samples collected at night, but the lab in the facility is closed, it will delay results, and we will not know promptly [enough] (Q8C1)”. According to the requirement of fast diagnosis, one suggested, “Can we assign the on-duty milking parlor supervisor to take responsibility? The parlor supervisor surely has some awareness of aseptic procedures and would be in the milking area nearly all the time, so there is no concern about sample contamination and on-time sampling (Q8B1)”. Although sampling and diagnostic procedures differ among companies, current procedures need to be amended to address the need for rapid diagnosis.

Interpretation of bacteriological culture results is also important for accurate diagnosis. Traditional culture methods need various tests to determine the pathogen, whereas rapid diagnosis kits provide faster and relatively easier interpretation. Participants insisted specialty training to perform interpretation would improve reliability of tests, as one participant suggested, “specialized individuals ensure result stability (Q9C1)”.

Addressing their concerns about rapid diagnosis tools, one participant suggested, “Can we explore the availability of rapid testing devices? It would be more convenient and help avoid sample contamination (Q9E1)”. Better solutions for diagnosis are needed for diagnosis of mastitis pathogens.

### 2.6. Strategies for Implementing Selective Treatments

Acknowledging current challenges and concerns, 93% of participants expressed a willingness to embrace the necessity of transitioning from blanket to selective treatments (Table 2). In providing insights into strategies to facilitate this shift, a commonly emphasized suggestion is the clear need to raise awareness among company executives. Participants widely consider “leadership awareness” as a pivotal factor in successful implementation of both selective treatments. One participant aptly noted, “Without leadership support, it is unlikely for anyone on the farm to dare to engage in selective treatments (Q3A2)”.

For both kinds of selective treatment, a change from the current blanket-treatment workflow is necessary, making staff training crucial. For SDCT, addressing issues such as use of teat sealants (100%) and proper dry-off procedures (85%) were indicated as key training priorities (Table 3). Furthermore, for STCM, accountability of staff (77%) and 24 h rapid diagnosis turn-around (69%) were key issues (Table 4). Therefore, comprehensive understanding of selective treatments should be a fundamental component of training to enhance performance.

Before initiating selective treatments, participants emphasized the need for a robust on-farm management system. As one participant suggested, “Selective treatments can’t be performed on poorly managed farms (Q4E1)”. Essential requirements include maintaining hygiene in housing areas and milking parlors, meticulous data collection encompassing DHI and disease history, and maintaining low bulk tank SCC to limit IMI prevalence.

Setting adequate herd-level standards based on circumstances is important for implementation of SDCT. The research group presented a purposed standard to participants and received varying levels of approval. Regarding herd selection, low bulk milk SCC (<150,000 cells/mL), a dry and cool climate, low CM incidence (<1.5% cows affected per month), and overall good teat-end scores was agreed by most of the participants (>65%). Other considerations included low prevalence and incidence of contagious pathogen mastitis (62%) and inorganic bedding (54%), whereas some suggested applying SDCT only in first-lactation heifers (62%). When selecting individual cows, the consensus among participants generally favored individual SCC history and CM records (Table 3). Additional factors under consideration included teat-end condition of individual cows (92%), cow-side examinations before dry-off to assess IMI status (77%), and milk yield (69%) (Table 3).

Implementing STCM requires clearly defined diagnostic criteria, whereas a veterinarian examining clinical signs and health records informs prognosis and treatment plans. Furthermore, 77% of participants agreed with establishing a standard to refine the diagnosis–treatment process and most (69%) acknowledged the importance of a 24 h rapid diagnosis turn-around (Table 4). For this, on-farm laboratories must prioritize culture testing turn-around times to enable selective decisions. Also, for some farms, rapid diagnosis kits would need to be adopted. Overall, for most farms, the process from diagnosis to treatment needs to be optimized to provide rapid turn-around.

In advocating for adoption of selective treatment, one participant stated, “I think the very first thing is to make the company leadership believe it is a meaningful thing to do (Q24A1)”. Having leaders being motivated would help farms to change. Participants also recommend selecting specific farms to conduct large-scale clinical trials, as one said, “If we can have several farms switch to selective treatment to test it, then we can know what will happen in real life (Q24C3)”. Although the discussion tried to cover every concern of participants, due to the lack of real-world experiences, it was expected that field trials would convince those with uncertainty. In addition, collaboration with specialists and timely guidance from universities would also be important, as one mentioned, “We can coordinate with universities like CAU to conduct this together. We can’t conduct this on our own (Q24B3)”.

## 3. Discussion

For group-owned dairy farms, upper management decisions will directly impact farm operations and workflows. To convince corporate leadership to adopt selective mastitis treatment plans, we must first understand and enable key motivators driving their decision-making. First, as public awareness around AMR grows, consumer values and preferences are increasingly influencing production practices [21]. It was recently reported that 79.8% of Chinese consumers are willing to pay premium prices for products that are produced without the use of antimicrobials [22]. Consumer-driven pressure on dairy companies shapes internal policies, promoting adoption of improved protocols at the farm level. By driving change through purchasing power, societal perspectives factor directly into dairies’ habits regarding AMU.

For managers in animal health, despite claims of general familiarity with AMR concepts, further discussion with the 15 participants revealed many knowledge gaps regarding specifics, particularly from a One Health perspective. Based on the results, there are many gaps in farm leadership’s knowledge surrounding AMR. Systematic education includes the detailed mechanism of emerged AMR, and its relation to daily farm practice may further enhance their motivation in reducing AMU in farm management. Similar training programs have been issued in other countries including the US which has been shown to be beneficial for farmworkers in knowledge and reduced antibiotics use [23,24]. Although the effects of training program on AMR need to be evaluated over a longer period [25], focused education to increase awareness of AMR risks is expected to positively shift attitudes towards behavioral change and reduce AMU on farms under their stewardship [26]. Governmental requirement would be another powerful force for changing AMU in the dairy industry. In the past two decades, the Chinese government published a series of announcements to control agricultural AMU [27]. Perhaps at least in part as a result of increased publicity, AMU in China’s agricultural sector decreased from 69,292 tons in 2014 to 30,903 tons in 2019, according to an official veterinary bulletin published by the Ministry of Agriculture and Rural Affairs [28]. It is expected that more calls from the government to reduce AMU would promote adoption of selective treatments.

Another motivation of participating Chinese dairy leaders to implement selective treatments is that selective treatment has potential economic benefits for dairy farms. For STCM, as CM inflicts substantial economic losses, averaging 1900 CNY (~US$270) per case, emerging resistance to intramammary antimicrobial therapies could further exacerbate costs. As reported, selective treatment of CM based on culture results can reduce AMU by >50% without sacrificing treatment efficacy [29]. With mastitis treatments accounting for a large portion of AMU on dairy operations, this represents substantial cost savings. Additionally, an average of 700 CNY (~US$100) worth of milk per cow is discarded annually due to antibiotics [30]. Selective strategies could drastically reduce discarded milk and revenue losses. However, assessments on the economic impacts of implementing selective treatments were not performed in China and economic outcomes could vary in different contexts. The cost of antibiotics used during the dry period ranges from US$10 to $28 per cow. While this may seem insignificant, it adds up when considering the number of cows needing treatment, making it worthwhile to save on these expenses. Previous studies in other countries have shown that SDCT can lead to better economic outcomes than BDCT [31].

Antibiotics are essential tools for mastitis treatment and control protocols. The efficacy of antimicrobials depends on their ability to prevent or impede growth of mastitis pathogens. Therefore, antimicrobial susceptibility tests can effectively guide treatment decisions [32], particularly for organisms and antimicrobials for which clinical breakpoints are available to interpret susceptibility testing. Based on our findings, large dairy farms in China rarely perform AMR testing to guide AMU. However, given the alarming rates of AMR in Chinese dairy herds [12,13], presumably AMR is reducing clinical and bacteriological cures of CM and effectiveness of DCT. Large-scale surveillance of AMR in dairy farms in China would provide data needed to determine appropriate antimicrobials for targeting mastitis pathogens as part of treatment guidelines.

The adverse effect of using less antibiotics on cattle health was also a concern of participants. Although no research has been conducted in China, STCM protocols have been adopted without adversely influencing bacteriological or clinical cures, SCC, milk yield, or incidences of recurrence or culling [17], and SDCT has been conducted in select herds without affecting udder health in many other countries [19]. Adapting selective treatments has potential to decrease AMU without adversely affecting cattle health under certain conditions.

As stated before, the workflow from diagnosis to treatment needs improvement. The added workload of DHI sampling may also burden farmers. To ease this transition, novel cow-side diagnostic tools could be introduced. For example, diagnostic tools like the Mastatest^®^ system are new, culture-based mastitis diagnosis tools that could be used, although they are not currently available in China [33]. Integrating user-friendly digital systems that facilitate interpretation of DHI data would similarly streamline shifting from conventional blanket therapy models by making selection more evidence-based.

Identifying herds that can apply selective treatments is a key factor affecting the success of these treatment strategies. In other countries where large-scale SDCT is practiced, it is recognized that no single standard fits all farms. Although basic criteria such as low bulk tank SCC and low incidence of CM are common, specific quantitative values vary across trials [19]. Participants’ suggestions aligned with these principles, emphasizing their agreement with core concepts while indicating specific requirements. However, these suggestions, though grounded in farm-level considerations, remain untested in the context of selective treatments. Currently, to our knowledge, there is no information available indicating adoption of either SDCT or STCM as a routine practice in China. Although some farms have independently piloted SDCT models, a lack of rigorous supervision and proper statistical validation hinders a clear demonstration of their value. On-farm studies, employing standardized selective treatment protocols could serve as a robust means to generate evidence to promote wider adoption while identifying and addressing practical challenges. Establishing detailed guidelines for implementation of integrated selective treatment regimens could catalyze a shift from conventional blanket approaches, fostering more prudent AMU practices in the Chinese dairy industry.

The focus group and questionnaire design intentionally guided discussions towards selective treatment concepts, rather than neutrally eliciting participants’ unbiased attitudes and practices. Furthermore, the observational results reflect stated opinions that may differ from actual on-farm behaviors, and front-line workers’ perspectives likely diverge from those of managers included here. Follow-up research should involve more open-ended elicitation of management practices from a broad range of farm personnel. Collecting quantitative AMU and AMR data and modeling effects of selective regimen implementation would also strengthen the evidence base.

## 4. Materials and Methods

This study was reviewed and approved by the China Agricultural University Research Ethics Board (Protocol CAUHR20240307). This manuscript was written according to the Consolidated Criteria for Reporting Qualitative Research (COREQ) framework [34].

### 4.1. Participants

This study was conducted with participants attending an in-person conference on *Selective Antimicrobial Treatments in the Chinese Dairy Industry* (28–29 October 2023) and their professional networks. The conference included 15 technical managers from the 13 largest dairy groups (based on the number of cows) in China which collectively represent 20.4% of the volume of milk produced in China. The Chinese dairy industry is primarily large herds, with cows housed in free-stall barns, and owned by companies that provide veterinary services and milk quality oversight. Many of the invited dairy groups owned >20 farms where they were responsible for treatment protocols and diagnostic standards. Technical managers monitor disease incidence on dairy farms and dictate management and treatment protocols.

At the start of the study, all participants consented to the study and expressed their permission for anonymized data to be used.

### 4.2. Procedure

A focus group discussion was conducted on 28 October 2023, to gain qualitative insights into research questions. The focus group discussion was hosted by J.G., Ph.D., male, Associate Professor at China Agriculture University. The 15 technical managers were randomly allocated into 3 groups for the focus group discussions. Before the focus group, participants were provided with introductions to key concepts regarding AMR and selective mastitis treatments to establish baseline understanding. The facilitator structured the focus group to directly address participants’ perceived needs and concerns regarding mastitis management approaches, clarify detailed definitions on terminology, explore expected barriers to implementing changes, and elicit suggestions on practical ways to facilitate adoption of selective treatment protocols. The focus groups lasted for ~3 h.

During the focus group, there was real-time data collection using questionnaires delivered through an online survey platform, WJX (www.wjx.cn, accessed on 28 October 2023) on core questions to assess detailed needs, standards, and solutions. Participants accessed the survey on their mobile devices by scanning a QR code. Facilitators assisted participants with the technology and answered questions as needed.

### 4.3. Data Collection

Qualitative data were collected during the focus group discussion through audio recordings that were retrospectively transcribed and analyzed. Questionnaire data were analyzed using descriptive statistics. The online WJX survey platform compiled results indicating the number and proportion of participants that selected each questionnaire response option.

### 4.4. Data Analyses

Transcripts of audio recordings were translated into English and imported into NVivo (QSR International, Version 14, Denver, CO, USA). Transcripts were divided into 4 parts based on questions provided for focus group discussion, including awareness of AMR and selective treatment, major concerns for SDCT, major concerns for STCM, and strategies for implementation of selective treatments. Oral responses and discussions of 3 groups were independently analyzed and labeled by 2 researchers (Y.X. and J.Y.) into sub-themes. The responses quoted from the transcript were identified using a structured system: “Q” denoted the question number, “A to E” represented individuals, and “1 to 3” indicated groups. Results were compared and discussed between 2 interpreters. Results of the questionnaire output by WJX were rearranged according to the main sections of visualization.

## 5. Conclusions

This study captured perspectives of Chinese dairy industry managers regarding AMR, SDCT, and STCM, and is a first step in identifying the challenges and obstacles of implementing selective treatment in China. Despite differing views on AMR and selective treatments, technical managers were generally supportive of adopting selective treatments. However, they highlighted the need for more evidence and tools, including the economic impacts, effects of delayed treatment, accurate milk recording interpretation, safe use of teat sealants, and pathogen spread. The findings revealed key challenges and barriers currently impeding selective AMU practices. These insights could inform efforts to promote judicious AMU on farms through targeted treatment regimens, reducing mounting selective pressure driving resistance. Field experiments on selective treatment regimens such as SDCT and STCM will be important to determine the efficacy of these regimens and will also be essential to convince the Chinese dairy industry that using these selective treatments can reduce AMU.

## Figures and Tables

**Table 1:** Background information on the 13 dairy groups of the 15 participants.

Dairy Group	Total Milk Yield (×10^3^ Tonnes)	No. Farms	No. Cattle (×10^3^)	No. Lactating Cows (×10^3^)	Average Annual Milk Yield per Cow (×10^3^ kg)
1	1989.7	73	416.2	204.5	11.4
2	1610.0	33	353.8	184.3	11.3
3	657.5	81	126.8	77.3	8.5
4	640.0	19	125.0	56.0	11.5
5	601.2	33	123.0	60.2	10.3
6	506.1	27	81.2	41.9	11.5
7	449.6	25	89.5	42.9	10.5
8	235.7	6	57.7	24.6	12.2
9	234.6	15	39.7	20.4	12.3
10	216.6	12	41.9	22.7	10.6
11	212.2	18	48.0	20.2	10.5
12	182.5	13	35.0	18.3	10.0
13	171.3	6	24.8	14.6	11.8
Total	7707.0	361	1562.6	787.9	

**Table 2:** Participants’ awareness of antimicrobial resistance (AMR) and selective treatments.

Questions	% of Participants
Are you familiar with AMR?	100% Yes
What AMR issue(s) is/are of concern?	100% AMR on farms causes antibiotic treatments to be ineffective 62% AMR and its negative impact on human health 54% Spread of AMR genes across human and animal-related pathogens 54% Antibiotic residues in milk 23% Antibiotic residues in the environment
What influence will the increased AMR bring to dairy groups?	92% Affect product quality 85% Lower consumer recognition 85% Cost increase (labor, products, discarded milk) 54% Adding difficulty to promoting organic products
Are you aware of selective dry cow therapy?	100% Yes
Have you tried SDCT on farms?	31% Yes 69% No
Are you aware of selective treatment of clinical mastitis?	38% Yes 62% No
Is it necessary to apply selective treatments in Chinese dairy herds?	93% Yes 7% No

**Table 3:** Major concerns regarding selective dry cow therapy.

Questions	% of Participants
Is DHI testing currently conducted within the group?	61% Performed on all farms 38% Performed on some farms
Is DHI currently being used for mastitis management?	69% Would prefer other data if available 69% Need a protocol to analyze DHI data to capture the information 31% Would rely on DHI data to adjust management strategies
Is DHI is useful for selecting cows SDCT?	92% Reliable 8% Not sure
Should teat sealant should be mandatory for SDCT?	69% Teat sealants are a must-have 31% Teat sealants are a must-have, but protection with antibiotics is still crucial in the dry period
Does your affiliated group currently use teat sealants for DCT?	100% Yes
Do you have problems with teat sealants?	92% Overpriced 77% Residues in milk 54% Extra teat stimulation
What key factors affect success of SDCT?	100% Use of teat sealants 92% Leadership is aware 92% Integrity of DHI data 92% Hygienic dry cow housing 85% Proper dry-off procedures 85% Accurate disease records 54% Gradual drying-off
What factors are important to select herds for SDCT?	92% Bulk tank SCC 77% Climate (dry and cold) 77% Low incidence of clinical mastitis (<1.5% per month) 69% Good teat-end scores 62% No contagious pathogens 62% Heifers only 54% Use inorganic bedding (e.g., sand)
What factors are important for to select cows for SDCT?	92% Teat-end [hyperkeratosis] scoring 77% Cow-side examination result 69% Milk yield

**Table 4:** Major concerns regarding selective treatment of clinical mastitis.

Questions	% of Participants
What are concerns for not treating Gram-negative and culture-negative moderate and mild cases?	69% Mild and modest cases becoming severe or culling rate increasing 62% Increasing bulk tank SCCs 46% Economic balance between cost of rapid testing and saving on antibiotics 92% Increased culling rate 69% Increasing nonfunctional quarters 69% Transmission of contagious pathogens 69% Decreased milk yield
What measures could be taken to reduce negative consequences of not treating ill cows?	77% House separately and milk last 77% Culling them on time 69% Use NSAIDs 62% Modify nutrition to increase herd immunity 69% Increase housing comfort 69% Dry-off earlier
What are current methods for diagnosis of mastitis?	31% Chromogenic culture-based diagnosis 46% Do not do culture, used third-party lab for bacteriological analysis 23% Traditional culture, including Gram stain, catalase, coagulase, and biochemical identification
What is the percentage of reported sample contamination?	23% >20% 38% 5–10% 15% 0–5% 23% Contamination not reported
What is the key factor in adapting rapid diagnosis tools effectively?	92% Aseptic sampling 77% Designated staff to interpret results 69% Adding “contamination” to results 62% Well-equipped farm lab
What do you think needs to be done to implement STCM?	92% Train staff on sampling and diagnosis 85% Increase awareness of executives 77% Clear standard for the entire process 69% Rapid (24-h) diagnosis turn-around 62% Refine farm management

## Data Availability

The data are available from the corresponding author on reasonable request.
